# Development and initial validation of the Orthotic Patient-Reported Outcomes—Mobility (OPRO-M): An item bank for evaluating mobility of people who use lower-limb orthoses

**DOI:** 10.1371/journal.pone.0293848

**Published:** 2023-11-02

**Authors:** Geoffrey S. Balkman, Alyssa M. Bamer, Phillip M. Stevens, Eric L. Weber, Sara J. Morgan, Rana Salem, Dagmar Amtmann, Brian J. Hafner

**Affiliations:** 1 Department of Rehabilitation Medicine, University of Washington, Seattle, Washington, United States of America; 2 Hanger Institute for Clinical Research and Education, Austin, Texas, United States of America; 3 Department of Physical Medicine and Rehabilitation, University of Utah, Salt Lake City, Utah, United States of America; 4 Gillette Children’s Specialty Healthcare, St. Paul, Minnesota, United States of America; 5 University of Minnesota, Minneapolis, Minnesota, United States of America; Iran University of Medical Sciences, ISLAMIC REPUBLIC OF IRAN

## Abstract

Lower limb orthoses (LLOs) are externally-applied leg braces that are designed to improve or maintain mobility in people with a variety of health conditions that affect lower limb function. Clinicians and researchers are therefore often motivated to measure LLO users’ mobility to select or assess the effectiveness of these devices. Patient-reported outcome measures (PROMs) can provide insights into important aspects of a LLO user’s mobility for these purposes. However, few PROMs are available to measure mobility of LLO users. Those few that exist have issues that may limit their clinical or scientific utility. The objective of this study was to create a population-specific item bank for measuring mobility of LLO users. Previously-developed candidate items were administered in a cross-sectional study to a large national sample of LLO users. Responses from study participants (n = 1036) were calibrated to a graded response statistical model using Item Response Theory methods. A set of 39 items was found to be unidimensional, locally independent, and function without bias due to characteristics unrelated to mobility. The set of final calibrated items, termed the Orthotic Patient-Reported Outcomes—Mobility (OPRO-M) item bank, was evaluated for initial evidence of convergent, divergent, and known groups construct validity. OPRO-M was strongly correlated with existing PROMs designed to measure aspects of physical function. Conversely, OPRO-M was weakly correlated with PROMs that measured unrelated constructs, like sleep disturbance and depression. OPRO-M also showed an ability to differentiate groups with expected mobility differences. Two fixed-length short forms were created from the OPRO-M item bank. Items on the short forms were selected based on statistical and clinical criteria. Collectively, results from this study indicate that OPRO-M can effectively measure mobility of LLO users, and OPRO-M short forms can now be recommended for use in routine clinical practice and research studies.

## Introduction

Lower limb functional impairments can result from a variety of neuromuscular and orthopedic health conditions. Individuals who experience lower limb muscle weakness or joint instability are often prescribed a leg brace, referred to as a lower limb orthosis (LLO). LLOs, like ankle-foot orthoses (AFOs), knee-ankle-foot orthoses (KAFOs), hip-knee-ankle-foot orthoses (HKAFOs), and functional electrical stimulation (FES) devices, are designed to compensate for functional or structural impairments, thereby improving the user’s balance and gait [1]. LLOs have been shown to improve mobility in adults with different health diagnoses, including stroke [2], spinal cord injury [3], and traumatic lower limb injury [4]. Despite the recognized benefits of LLOs, orthotists infrequently evaluate their effectiveness by measuring patients’ mobility outcomes [5, 6]. In a prior study, orthotists reported that time constraints and the lack of instruments designed for LLO users were major barriers to routine outcomes measurement [6].

Patient-reported outcome measures (PROMs) are self-report instruments used to collect and quantify individuals’ experiences of their symptoms, functioning, or other aspects of health. PROMs may be developed for people with a wide range of health conditions or specifically target a group of people, like LLO users, who have a specific health characteristic or share a common experience [7]. PROMs developed in recent years have benefited from the application of modern psychometric methodologies, like item response theory (IRT) [8]. IRT-based PROMs include item banks, which are sets of calibrated items (i.e., survey questions) designed to measure a trait (such as LLO users’ mobility) along a continuum [9]. Item banks are typically scored on a T-score metric that is referenced to the population mean. Because items ranked along the mobility continuum, an estimate of an individual’s level of the trait can be generated without administering all items in the bank. Fixed-length instruments that include only a subset of items from the larger item bank, referred to as “short forms,” can produce T-scores that highly correlate with the T-scores based on all the items in the item bank. Alternatively, items can be administered as a computerized adaptive test (CAT) [9]. CAT and Short forms can therefore be customized to include only a small number of clinically-relevant items and may address concerns about administrative burden and clinical applicability reported by orthotists previously [6].

An example of an IRT-based PROM that has been used to measure mobility-related outcomes in LLO users is the Patient-Reported Outcome Measure Information System—Physical Function (PROMIS-PF) item bank [10]. PROMIS-PF scores are centered on responses from a general population and may be challenging to interpret when assessing mobility of LLO users. DiBello et al. reported that people who received an AFO post-stroke had a mean PROMIS-PF T-score of 30.8—nearly two standard deviations below the general population mean [11]. It is challenging to accurately measure and evaluate changes in mobility with PROMIS-PF when LLO users are so near the floor of the scale (i.e., a T-score of 30.8 places someone within the bottom 3% of the US general population). The 20-item PROMIS-PF short form also includes many items that address self-care activities and upper limb movements that may not be affected by use of a LLO.

Other types of PROMs have also been administered to LLO users. One example is the Orthotic and Prosthetic User’s Survey–Lower Extremity Functional Status (OPUS-LEFS), developed for use with both prosthesis and orthosis users [12]. Despite the potential utility of a single PROM for evaluating two groups of device users, prior research has suggested that people who use LLOs may have unique experiences related to mobility [13, 14]. Another example is the Lower Extremity Functional Scale (LEFS), a survey intended for people with orthopedic injuries [15]. Although the LEFS has been used previously to evaluate LLO users’ perceived functional abilities [16, 17], it may not include activities and situations most relevant to the majority of LLO users. According to LLO users in prior qualitative studies [13, 18], multiple items included in PROMIS-PF, OPUS-LEFS, and LEFS describe activities and situations that are perceived as less important to mobility with a LLO. The content limitations present in these existing PROMs suggest that development of a new instrument for measuring mobility of LLO users may be warranted.

A pool of candidate items was developed to measure aspects of mobility relevant to LLO users in prior work [19]. The process followed recommendations established by the PROMIS network for developing candidate items for an IRT-based PROM [20]. Focus groups were first conducted with LLO users to learn how mobility was affected by use of an orthosis, examine applicability of a previously-published conceptual model of mobility [14], assess a proposed construct definition, and solicit examples of activities people performed with their LLO. “Mobility” was defined as moving intentionally and without the help of another person [19]. Candidate items were generated from analysis of focus group transcripts, a literature review of PROMs designed to measure lower limb mobility, and input from clinical and scientific experts. Members of a stakeholder advisory panel assisted with narrowing the pool of candidate items and selecting those that were deemed to be most suitable to measuring LLO users’ mobility. Lastly, cognitive interviews were conducted with LLO users to evaluate clarity and comprehensibility of each item, as well as the comprehensiveness of the content covered by all candidate items. These efforts resulted in a pool of 100 candidate items that addressed a wide range of activities and situations relevant to LLO use [19].

The purpose of this study was to develop the Orthotic Patient-Reported Outcomes–Mobility (OPRO-M), a self-report item bank for measuring mobility of LLO users. To achieve this goal, the previously-developed candidate item pool [19] was administered to a large national sample of LLO users. Results were used to identify items that fit an IRT model and could be included in the OPRO-M item bank. Initial evidence of validity was also examined to assess the psychometric quality of the bank. Fixed-length short forms were created to encourage adoption of OPRO-M by clinicians and researchers.

## Methods

### Participants

LLO users were recruited with flyers displayed in orthotic clinics across the U.S., notices posted to social media, and emails and texts sent to individuals who had previously received orthotic services. Selection criteria were established to include adults with chronic lower limb impairments who used orthoses extending proximally from the foot to a level above the ankle. Eligible participants were 18 years of age or older; were able to read, write, and understand English; did not require help from another person to move from one place to another; were prescribed an ankle-foot orthosis (AFO), knee-ankle-foot orthosis (KAFO), hip-knee-ankle-foot orthoses (HKAFO), or functional electrical stimulation (FES) device for one or both legs; and had at least six months of experience using an orthosis. Individuals with a major upper and lower limb amputation were considered ineligible for the study.

A sample size of 1000 participants was targeted to conduct the necessary analyses, including IRT modelling [21] and differential item functioning (DIF) evaluation. Convenience sampling was supplemented with targeted recruitment to ensure that select subgroups of participants included a sufficient number of participants for DIF analyses. A sample of at least 200 per group is recommended for DIF detection [22, 23], and with 1000 participants, we expected to have adequate subsample sizes to evaluate DIF by gender (i.e., man, woman), age (i.e., <65 years, >65 years), clinical condition type (i.e., orthopedic, neuromuscular), and etiology (i.e., traumatic, non-traumatic). Recruitment targets of 30 or more participants were also set for groups of people with less common, but clinically-important characteristics. These included people who use specific types of LLOs (e.g., unilateral and bilateral KAFOs) and people with specific health conditions (e.g., post-polio syndrome, traumatic brain injury, muscular dystrophy).

### Procedures

A cross-sectional study was conducted to administer the candidate items to a national sample of LLO users. Participants completed online screening questions or spoke with a research staff member to determine their eligibility prior to taking the survey. Responses to the survey were collected and managed using Research Electronic Data Capture (REDCap) software hosted at the University of Washington [24]. Paper copies of the survey, along with a pre-paid return envelope were made available upon request. The survey included candidate items for measuring LLO mobility [19], PROMs developed previously to measure constructs both related and unrelated to mobility, and questions about demographics, health conditions, and orthosis use. All surveys were reviewed for missing data or irregularities and follow-up phone calls were made to collect missing data or clarify inconsistent responses. Two investigators examined completed surveys with invalid contact information or incorrect responses to quality assurance questions and reached consensus on whether to include the records in the final dataset. All procedures were reviewed by the University of Washington Human Subjects Division Institutional Review Board and determined to meet requirements for exempt status. Respondents provided informed consent by reading the information statement and subsequently starting the online survey or paper survey.

### Measures

The 100 items from the candidate item pool that all began with the context, “Are you currently able to…” were administered to assess a person’s mobility at the time of assessment. The response options were “without any difficulty” (5), “with a little difficulty” (4), “with some difficulty” (3), “with much difficulty” (2), and “unable to do” (1) [19]. Candidate items were divided randomly into five sets of twenty items and arranged by expected level of difficulty. The electronic survey also randomized the order in which the five sets of candidate items were administered to limit the effects of respondent fatigue on data quality.

Several PROMs that have been used previously to evaluate mobility and/or physical functioning in LLO users were included in the survey to evaluate convergent construct validity of the OPRO-M item bank. These included the PROMIS-PF 20-item short form [10], OPUS-LEFS [12], and LEFS [15]. PROMIS-PF has been shown to have evidence of validity when tested with LLO users [25] and evidence of validity and/or reliability when tested with people diagnosed with a variety of health conditions that affect the lower extremities [26–29]. OPUS-LEFS has been shown to have excellent internal consistency when tested with a mixed sample of orthosis and prosthesis users [12]. LEFS has demonstrated desirable measurement properties when administered to people with orthopedic disorders [30] and those affected by stroke [31].

The Patient-Reported Outcome Measure Information System– 29-item short form version 2.0 (PROMIS-29) [32] was included to evaluate divergent construct validity of OPRO-M. PROMIS-29 includes four items each from seven PROMIS domains, and one 11-point numeric pain intensity rating scale. Evidence of reliability and validity of PROMIS-29 has been demonstrated in a representative U.S. national sample [33].

Questions about demographics (e.g., age, gender, race and ethnicity, military status), health (e.g., diagnosis, comorbidities, fall history), orthosis type (e.g., orthosis level, laterality), orthosis use (e.g., history of use, typical weekly and daily use), and assistive device use (e.g., types of devices used, frequency of use) were included in the survey to characterize the study sample.

### Data analysis

Analyses were conducted in multiple steps, following instrument development guidelines used by the PROMIS network [34]. First, we reviewed the data for quality and checked for missing data. Second, to calibrate the item bank to an IRT model, we completed analyses to verify that the required assumptions (i.e., unidimensionality, local independence, and monotonicity) of IRT modeling were met. Third, items were calibrated to an IRT model and evaluated for differential item functioning (DIF). Fourth, fixed length short forms were created. Lastly, the reliability and initial construct validity of the item bank and short forms were evaluated. Analyses were conducted using MPlus (unidimensionality) [35], IRTPRO (IRT modeling and local independence) [36], R (DIF) [37], and Stata (validity analyses and sample descriptive statistics) [38] software.

#### Data quality and missingness review

Survey responses were first screened for quality. Three quality assurance (QA) questions that asked respondents to select specific response options were inserted into the survey (e.g., “Please select ’with much difficulty’ as a data check”). Surveys with two or more incorrect responses to QA questions were removed from analyses. Surveys with only one incorrect QA response were examined further for patterns of inconsistency. Data quality was also reviewed by comparing responses to multiple mobility-related items that described similar activities (e.g., getting into and out of a car). Two investigators reviewed surveys with responses to similar items that differed by three or more response categories. Surveys with multiple inconsistencies were removed from subsequent analyses. Missingness was evaluated by calculating the percentage of missing responses for each OPRO-M item. Items with 0.5% or more missingness were considered for removal.

#### Confirmation of IRT assumptions

Local dependence (LD) was evaluated using Chen and Thissen’s LD index to verify responses to one item were independent of responses to another [39]. Pairs of items with an LD *X*^2^ statistic greater than 10.0 were flagged and reviewed. Items that were frequently flagged for LD and had lower discrimination and/or less clinical importance were considered for removal. An iterative approach was used to review items as a team, remove those items with higher LD and less clinical utility, and rerun the analysis. The assumption of local independence was determined to be met when all item pairs had an LD *X*^2^ statistic of less than 10.0. A one-factor confirmatory factor analysis (CFA) was completed to assess whether the items were unidimensional (i.e., measured a single primary construct) using the weighted least square mean and variance-adjusted estimator in MPlus [35]. The IRT assumption of unidimensionality was determined to be met if the model fit statistics (i.e., comparative fit index (CFI) and Tucker-Lewis Index (TLI)) were 0.90 or higher, and the misfit statistic (i.e., root mean square error of approximation (RMSEA)) was 0.08 or lower [40]. The assumption of monotonicity was evaluated by calculating Lovinger H coefficients and examining item trace lines. Acceptable monotonicity was indicated when H coefficients were at least 0.3 for individual items and 0.5 for the overall scale [41], and all items’ cumulative trace lines had increasing patterns.

#### IRT calibration and DIF analyses

After removing items with significant LD, remaining items were calibrated using Samejima’s graded response model [42]. Calibration of the items produced estimates of the difficulty and discrimination item parameters. Item response curves generated after fitting the final model were visually inspected to evaluate if the response options functioned well for the calibrated items. Final calibrated T-scores were centered on the representative study sample with a mean of 50 and standard deviation of 10. Reference tables were generated to present means, quartiles, SD, and ranges for the total sample and for participants with different types of paresis (i.e., spastic, flaccid, or no paresis). Additional tables were created for subsample comparisons. The means and standard deviations were also used to plot cumulative distribution functions that can be used to estimate percentiles based on T-scores.

DIF analyses were performed using the *lordif* program in R [43]. Presence of DIF indicates that two people with the same level of mobility have different scores because of some other unrelated factor. DIF was evaluated with respect to gender (men vs. women), age (under 65 years vs. 65 years and older), clinical condition type (orthopedic vs. neuromuscular), and clinical condition etiology (traumatic injury vs. non-traumatic condition). Items were flagged as having statistically significant DIF when a change in McFadden’s pseudo R^2^ statistic of 0.13 [23] or a 5% change in β coefficients [44] was observed. Items with statistically significant DIF were evaluated for potential exclusion from the item bank based on their overall impact on T-scores.

#### Development of short forms

Subsets of items from the item bank (i.e., short forms) were developed for use in both clinical and research applications. Short forms are intended to be brief and easy to administer either on paper or computer. An interactive item selection tool was developed in Microsoft Excel [45] to maximize coverage along the mobility continuum and the reliability of short forms. The selection tool included information about item reliability (i.e., item information), difficulty (i.e., location along the trait continuum), reading level (based on Flesch-Kincaid Grade Level [46]), and content. A reading level of 8^th^ grade or below was targeted [47]. We used the selection tool to compare the reliability and range of measurement of proposed short forms of various lengths relative to the full item bank. Items selected for short forms balanced important clinical content with high reliability, lower reading level, and burden of administration (i.e., length of short form). Advisory panel feedback was used to identify which of the proposed short forms were most suitable for generic public use. Lin’s concordance correlation coefficient (CCC) was used to ensure scores generated from the proposed short forms had strong agreement with scores from the full item bank [48]. A CCC of 0.95 or higher was considered ideal and interpreted as substantial agreement [49]. Short form summary score to T-score scoring tables were generated using IRTPRO [36].

#### Evaluation of initial reliability and validity

Reliability of scores along the mobility continuum was evaluated using the test information function graphs for individual items and the overall bank. The test information graphs can be converted to classical test theory-based reliability estimates which allows for examination of reliability across the scale continuum [50]. Scale information of 10 corresponds to reliability of 0.9 [51]. The effective range of measurement was determined by calculating the range of scores above this threshold. The effective range of measurement and participants’ responses were visually compared by plotting the histogram of responses against the scale information functions. We aimed for the effective range of measurement of the OPRO-M item bank to range approximately from 3 SD below to 3 SD above the mean.

Floor effects were evaluated by examining scores from OPRO-M short forms in subgroups with characteristics related to lower mobility (i.e., participants with bilateral orthoses who used assistive devices in their homes). Similarly, ceiling effects were evaluated by examining scores in subgroups with characteristics related to higher mobility (i.e., those with unilateral AFOs who never used assistive devices). Floor and ceiling effects were indicated when 15% or more of the subgroup scored at the lowest or highest end of the scale, respectively [52]. We hypothesized that floor and ceiling effects would not be present for OPRO-M short forms.

Known-groups construct validity, which examines differences in scores between people with different levels of a trait, was evaluated by comparing OPRO-M scores across groups of LLO users expected to exhibit increasing levels of mobility. A one-way analysis of variance (ANOVA) was used to evaluate differences in OPRO-M scores. Tukey post-hoc pairwise comparisons were also performed to identify which subgroups were significantly different (p<0.05). We hypothesized that OPRO-M scores would be higher for participants with fewer comorbidities, fewer falls (in the past 12 months), less reliance on assistive devices, lower levels of bracing, and less severe spasticity.

Convergent construct validity, which examines correlations between instruments that measure more related constructs, was evaluated by comparing OPRO-M scores to OPUS-LEFS, LEF, and PROMIS-PF scores. The Shapiro-Wilk test [53] was used to evaluate normality and linearity of each distribution, and when both comparison measures were normally distributed, Pearson’s correlation coefficients was calculated. When either comparison measure was non-normally distributed, Spearman’s rank correlation coefficient was used. We hypothesized that strong correlations (i.e., r_s_ ≥ 0.7) [54] would be identified between OPRO-M and each of the other instruments given that they measure very similar constructs (i.e., mobility and/or physical function) and are similar types of measures (i.e., PROMs).

Divergent construct validity, which examines correlations between instruments that measure less related constructs, was evaluated by comparing OPRO-M scores to scores from instruments included in the PROMIS-29 that measure constructs less related to mobility. The analysis procedures were the same as the convergent construct validity analysis. We hypothesized that moderate correlations (i.e., 0.4 ≤ |r_s_ | < 0.7) [54] would be identified between OPRO-M and PROMIS domains that are likely to be related to mobility, including Ability to Participate in Social Roles and Activities, Fatigue, and Pain Interference. We also hypothesized that weak correlations (i.e., |r_s_ | < 0.4) [54] would be identified for PROMIS domains that are less likely to be related to mobility, including Anxiety, Depression, and Sleep Disturbance.

## Results

### Data quality and missingness

**Fig 1:**
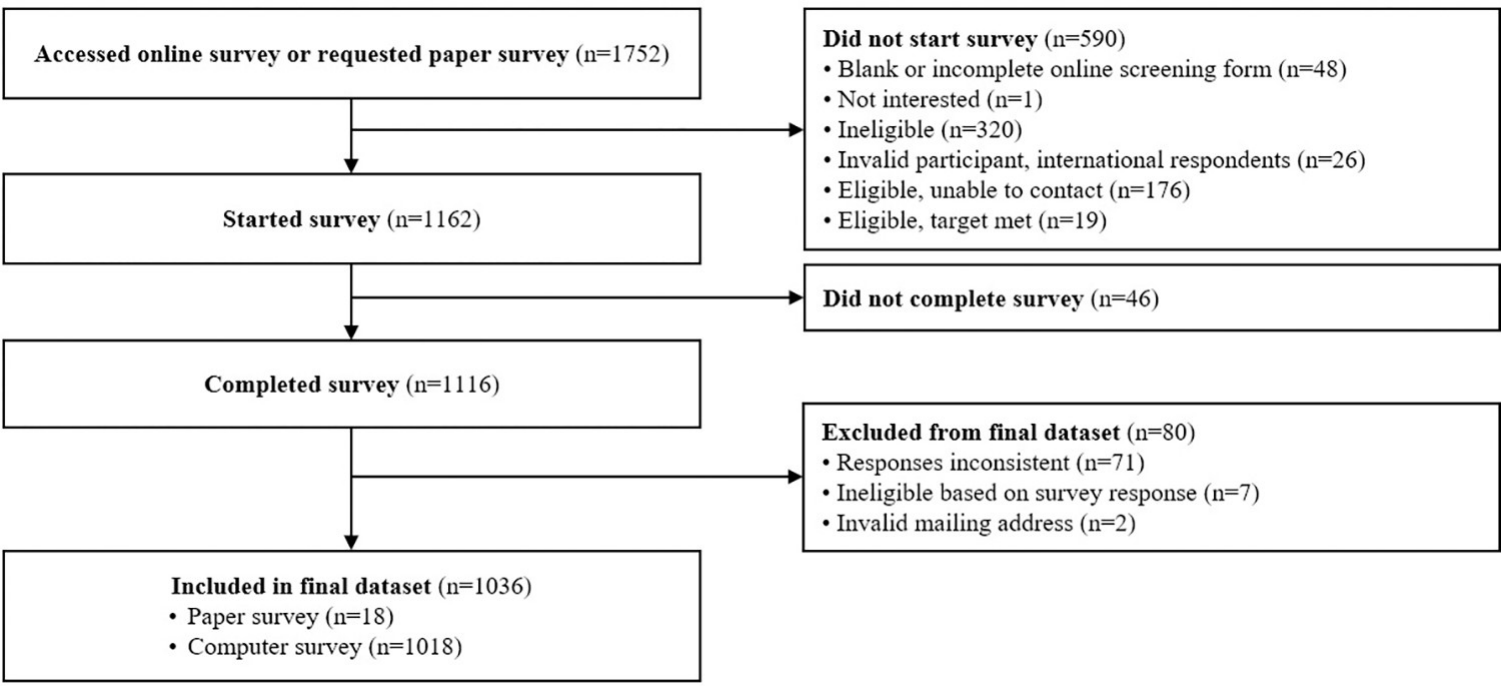
Potential participants were removed from the study sample for a variety of reasons. The large majority of surveys were completed electronically.

Data were collected from March to September 2021. A total of 1,116 participants completed the survey. Most participants completed the survey online, but a small number of participants (n = 18) completed a paper version of the survey. Responses from 20 participants were removed from analyses due to two or more incorrect responses to quality assurance questions. Responses from another 51 participants were removed due to numerous inconsistencies in their responses. Nine records were excluded due to ineligibility based on responses or invalid mailing addresses. Responses from 1036 participants were included for subsequent analyses (Fig 1). Missingness for the OPRO-M candidate items in the final dataset ranged from 0% (no missing responses for 54 items) to 0.48% (5 missing responses for one item).

Just over half of all participants were women and the mean age was 60 years (median: 62 years; range: 19–94 years). The most frequently reported health conditions were peripheral neuropathy, traumatic lower limb injury, spinal cord injury, non-traumatic lower limb condition (e.g., osteoarthritis), Charcot-Marie-Tooth disease, stroke, and multiple sclerosis. A quarter of the sample (n = 257) reported having more than one condition that affected their lower limb mobility. Nearly 90% of participants used AFOs and 89% used their orthosis(es) at least four days per week. Additional sample characteristics can be found in Table 1.

**Table 1:** Sample characteristics.

*Characteristic*	*n*	*%*
*Gender*	* *	* *
Woman	520	50%
Man	514	50%
Other	2	<1%
*Age*		
18–39 years	111	11%
40–64 years	474	46%
65 or more years	451	43%
*Ethnicity*		
Hispanic or Latino	33	3%
Not Hispanic or Latino	944	91%
Unknown or prefer not to answer	59	6%
Race*		
American Indian or Alaskan Native	1	1%
Asian	17	2%
Black or African-American	44	5%
Native Hawaiian or Pacific Islander	1	0%
White	932	91%
Multiple races	16	1%
Unknown or prefer not to answer	25	2%
*Military status*		
Servicemember	2	<1%
Veteran	127	12%
Not Servicemember or Veteran	907	88%
*Geographical region*		
West	305	29%
Midwest	261	25%
Northeast	141	14%
South	329	32%
*Health condition* ^a^		
Peripheral neuropathy	231	22%
Traumatic orthopedic LL injury	155	15%
Spinal cord injury	124	12%
Non-traumatic orthopedic LL condition	111	11%
Charcot-Marie-Tooth disease	97	9%
Stroke	90	9%
Multiple sclerosis	69	7%
Post-polio syndrome	65	6%
Muscular dystrophy	47	5%
Traumatic brain injury	43	4%
Non-traumatic spinal injury	39	4%
Other condition	177	17%
*Orthosis type*		* *
Unilateral AFO	649	63%
Bilateral AFO	281	27%
Unilateral KAFO	62	6%
Bilateral KAFO	16	2%
AFO and KAFO	12	1%
Unilateral HKAFO	4	<1%
Bilateral HKAFO	2	<1%
AFO and HKAFO	1	<1%
Unilateral FES	9	1%

LL: lower limb, AFO: ankle-foot orthosis, KAFO: knee-ankle-foot orthosis, HKAFO: hip-knee-ankle-foot orthosis, FES: functional electrical stimulation device

^*a*^ Respondents could select more than one option.

#### IRT assumptions

The LD analysis flagged 21 items for significant LD with at least 10 other items. After six rounds of item removal, a total of 61 items were eliminated due to LD. The remaining items exhibited no significant LD (median *X*^2^ = 1.9, range -1.8 to 9.6). Results of the CFA for the 39 items suggested that the items were unidimensional, indicated by adequate model fit (CFI = 0.971; TLI = 0.969; RMSEA = 0.057). Monotonicity was confirmed, as all item trace lines displayed increasing patterns, and the Lovinger H coefficient was 0.648 across all items, with individual items ranging from 0.587 to 0.693.

#### Calibration and DIF analysis

The graded response model was fit to the remaining 39 items, and item parameters (i.e., difficulty and discrimination) were generated. Discrimination parameters ranged from 1.63 to 3.23, and average difficulty ranged from -1.56 to 2.24. The response curves generally showed distinct peaks for the five response options, except for one item that described running on level ground, that functioned as an item with dichotomous response options. The item was retained with all five response options to maintain consistency in response option format. No significant DIF was identified between any comparison groups using the R^2^ criterion. One item that described standing up from a chair without using armrests displayed minor DIF by gender, using the β cutoff criteria, but was retained because the item-level DIF had minimal impact on instrument-level DIF, and the described activity was deemed as clinically important.

#### Short forms

**Fig 2:**
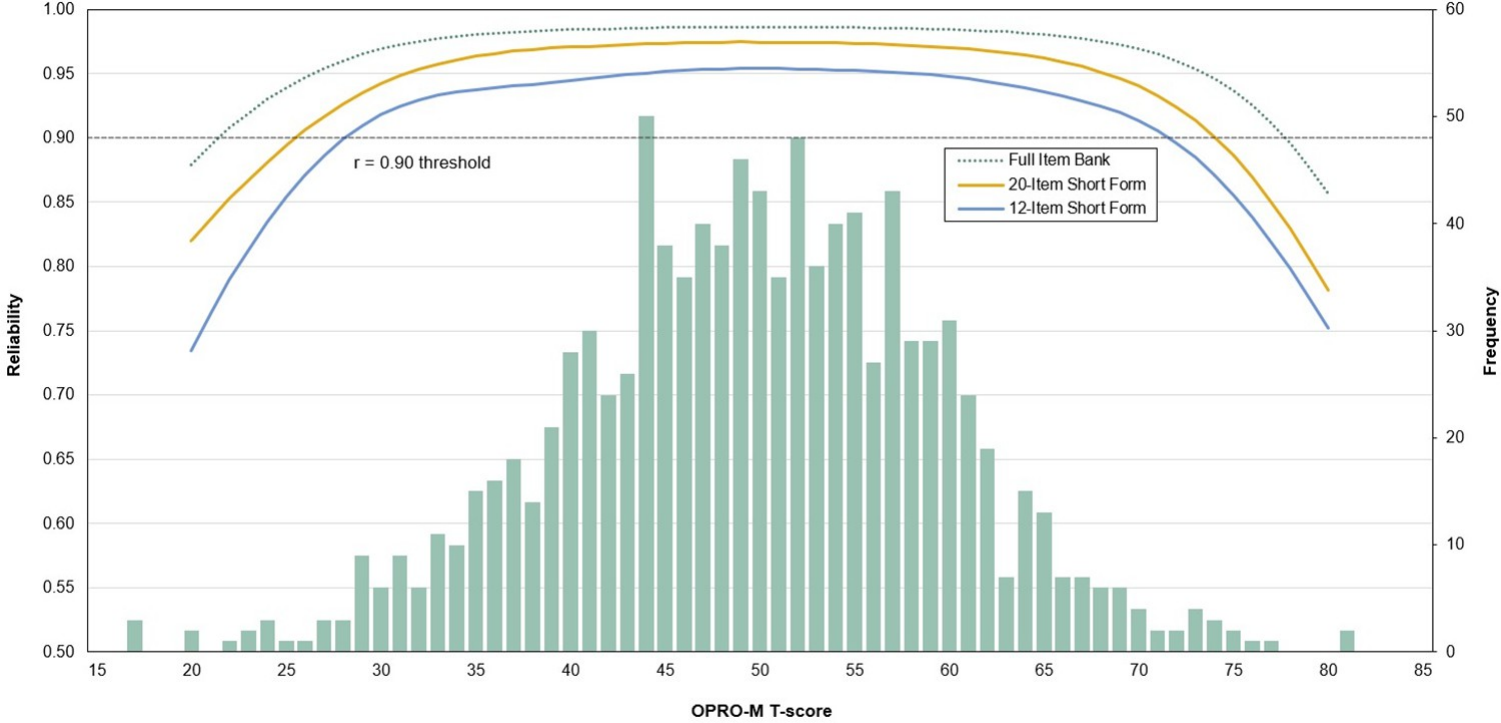
Reliability of the OPRO-M item bank and the 20- and 12-item short forms. The effective range of measurement (> 0.90) is nearly 2.75 SD below and above the mean for the full item bank, nearly 2.50 SD below and above the mean for the 20-item short form, and nearly 2.25 SD below and above the mean for the 12-item short form.

Four investigators independently used the item selection worksheet to identify abbreviated sets of items that included items with higher discrimination, clinical utility, and readability. Items that were selected through consensus were used to develop fixed-length short forms for clinical and research applications. Candidate 20-, 12-, 6-, and 4-item short forms, and their respective reliability statistics, were presented to the advisory panel for review. The panel members agreed that the 12- and 20-item short forms were most practical for clinical and research applications. The short forms showed acceptable measurement precision (standard error less than 3.0) from a T-score of 31.6 to 64.7 for the 12-item short form, and from 28.1 to 70.0 for the 20-item short form (see Fig 2). T-scores obtained from the OPRO-M short forms were highly correlated with T-scores obtained from the complete 39-item bank (12-item short form: ρ_c_ ≥ 0.968, 95% CI: 0.965–0.972, p < 0.001; 20-item short form: ρ_c_ ≥ 0.987, 95% CI: 0.985–0.988, p < 0.001).

The reading level for all except one of the 39 items was at or below the targeted 8^th^ grade level (median grade level 5.8, range 3.6 to 9.1). The item with a 9.1 grade reading level described doing heavy outdoor work and included the example of using a long-handled shovel. Readability statistics were similar for the 20-item short form (median grade level 5.8, range 3.6 to 7.8), and lower for the 12-item short form (median grade level 4.9, range 3.6 to 6.2). Final versions of the short forms and their corresponding scoring tables were created for public dissemination. Reference tables were generated for the following subsamples: gender (i.e., men, women), age (i.e., under 65 years, 65 years and older), orthosis type (i.e., unilateral AFO, bilateral AFO, unilateral or bilateral KAFO), and comorbidities (i.e., 0, 1, 2 or more comorbidities). The reference tables and scoring instructions were included in a user guide. The items included in the OPRO-M v1.0 item bank and their parameters can be found in Table 2.

**Table 2:** The 39 items included in the OPRO-M item bank with their item parameters.

Item text, preceded by, “Are you currently able to…”	Included in	*Slope*	*Threshold*				*Average*
	short form	*a*	*b*_1_	*b*_2_	*b*_3_	*b*_4_	*difficulty*
walk a short distance in your home?	12, 20	2.29	-2.79	-2.01	-1.13	-0.30	-1.56
step over an extension cord?	20	2.23	-2.94	-1.74	-1.04	-0.16	-1.47
take 2 steps backwards?	12, 20	2.26	-2.15	-1.27	-0.41	0.48	-0.84
lift a bag of groceries from the floor?		2.27	-1.93	-1.19	-0.46	0.35	-0.81
sweep the floor?	12, 20	2.59	-1.61	-1.00	-0.39	0.29	-0.68
stretch to get a book from a high shelf?		1.97	-1.77	-1.05	-0.27	0.67	-0.61
walk without catching your toes on the ground?	20	1.65	-1.93	-1.05	-0.25	0.82	-0.60
step up and down curbs?	12, 20	3.06	-1.84	-0.98	-0.10	0.96	-0.49
walk while talking on a mobile phone?		2.31	-1.25	-0.80	-0.28	0.55	-0.45
walk while carrying a shopping basket in one hand?	20	3.13	-1.17	-0.70	-0.17	0.55	-0.37
get in and out of the back seat of a 4-door car?		2.04	-2.11	-0.76	0.24	1.32	-0.34
walk from one room to another in the dark?		2.57	-1.50	-0.72	0.02	0.90	-0.33
walk across a large parking lot?	12, 20	2.85	-1.49	-0.62	0.10	0.79	-0.31
step off of an escalator?		2.71	-1.46	-0.75	0.04	0.98	-0.30
walk without looking down at the ground?		2.14	-1.53	-0.68	0.06	0.97	-0.30
keep walking when people bump into you?		2.92	-1.44	-0.61	0.12	1.14	-0.20
walk over loose gravel?	12, 20	2.82	-1.66	-0.56	0.26	1.27	-0.17
stand up from a chair without using the arm rests?		1.99	-1.19	-0.45	0.17	1.12	-0.09
walk across a 4-lane road at a crosswalk before the light changes?	20	2.87	-0.99	-0.41	0.23	0.98	-0.05
walk in an area that is too crowded to see the ground ahead of you?	20	3.23	-1.26	-0.39	0.36	1.21	-0.02
adjust your step when a pet or child suddenly moves in front of you?		2.52	-1.67	-0.39	0.45	1.53	-0.02
walk between rows of occupied seats like those in a theater or church?		2.62	-1.52	-0.38	0.46	1.41	-0.01
walk on uneven grass?	12, 20	2.65	-1.58	-0.33	0.49	1.63	0.05
walk on an unlit street or sidewalk?	20	2.96	-1.11	-0.35	0.37	1.36	0.07
walk up stairs placing only one foot on each step?		2.09	-0.95	-0.29	0.33	1.21	0.08
regain your balance after you stumble?		2.28	-1.73	-0.33	0.61	1.78	0.08
step on and off a bus?		3.01	-1.18	-0.37	0.42	1.47	0.09
walk to your seat in a dark theater?	20	3.07	-1.11	-0.25	0.46	1.30	0.10
walk down hills?		2.65	-1.20	-0.11	0.69	1.67	0.26
walk quickly without stumbling?		2.79	-0.60	0.01	0.66	1.46	0.38
keep up with others when walking?	12, 20	2.70	-0.78	0.10	0.78	1.76	0.47
descend a flight of stairs in the dark?		2.72	-0.56	0.21	0.93	1.81	0.60
climb several flights of stairs?	12, 20	2.41	-0.56	0.29	0.95	1.85	0.63
walk across a slippery floor?		2.39	-0.80	0.32	1.07	2.00	0.65
do heavy outdoor work like digging with a long-handled shovel?		2.32	-0.23	0.35	0.95	1.68	0.69
carry a laundry basket up a flight of stairs?	20	2.70	-0.22	0.41	1.01	1.85	0.76
climb a flight of stairs without a handrail?	12, 20	2.82	-0.19	0.57	1.13	1.94	0.86
go for an all-day hike?	12, 20	2.10	0.72	1.39	1.93	2.68	1.68
run on level ground?	12, 20	1.63	1.35	1.97	2.51	3.13	2.24

#### Evaluation of initial construct validity

The test information function graphs showed that the OPRO-M item bank measured with high reliability (> 0.90) from more than 2.75 SD below to 2.75 SD above the mean. The effective range of measurement for the 20-item short form was 2.50 SD below to nearly 2.50 SD above the mean, and the range for the 12-item short form was nearly 2.25 SD below and above the mean.

No floor effects were identified for the 134 participants with bilateral orthoses who used assistive devices in their homes. Only 2.2% and 3.7% of the bilateral subsample reached the minimum score on the 20- and 12-item short forms, respectively. Further, only 2.3% scored within the lowest three scores for the 20-item short form, and 6.7% for the 12-item short form. Similarly, no ceiling effects were identified for the 321 participants with unilateral AFOs who never use assistive devices. Only 0.6% of the unilateral subsample (2 participants) reached the maximum score for both short forms, and only 1.3% and 4.1% of these participants scored within the highest three scores for the 20-item and 12-item short form, respectively.

**Fig 3:**
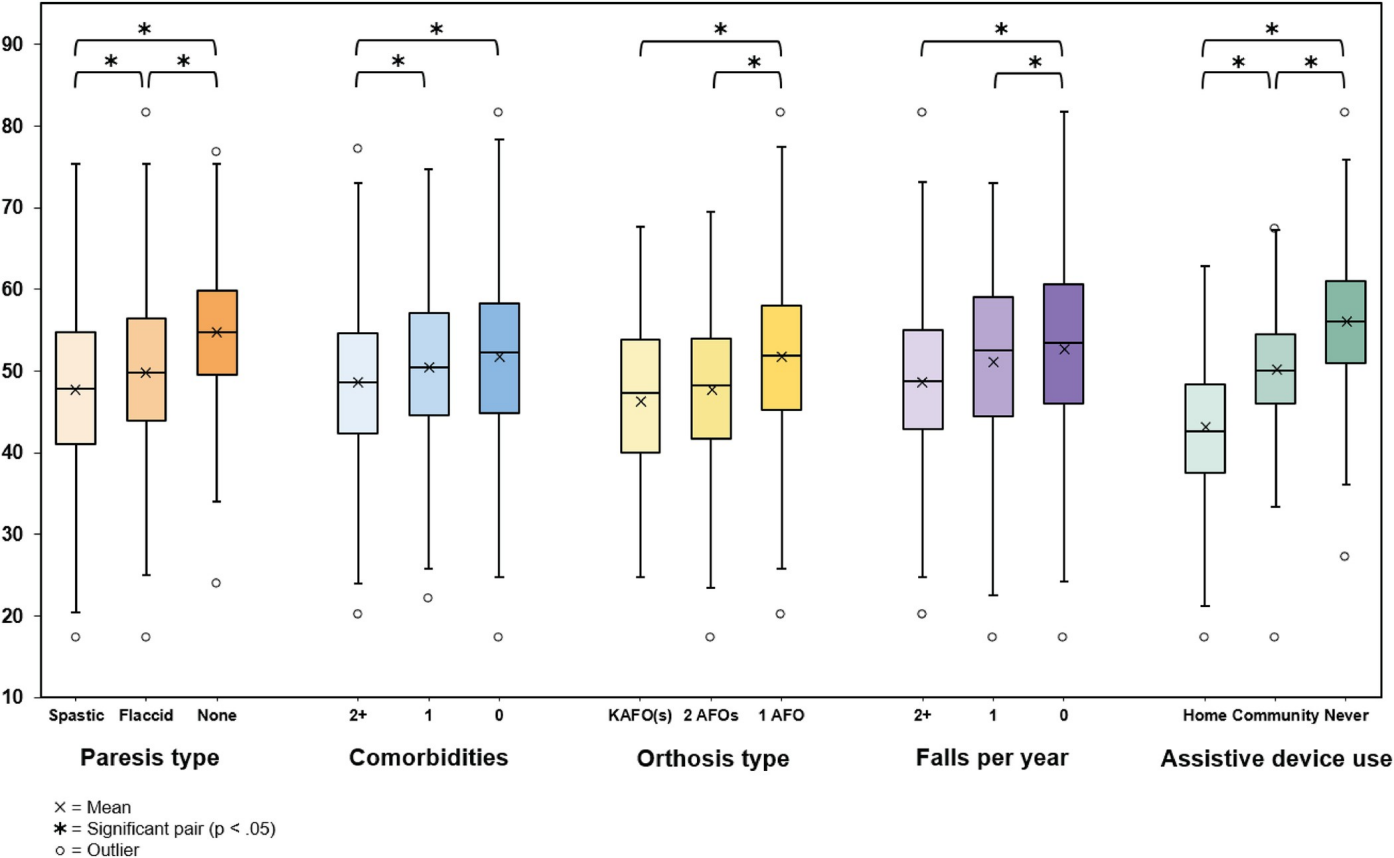
Results of the known groups construct validity analysis. Differences between means of all three comparison groups were statistically significant for paresis type and assistive device use. Differences between means of two of the three groups were statistically significant for number of comorbidities, orthosis type, and number of falls in the past 12 months.

One-way ANOVA testing revealed that there were statistically significant differences in OPRO-M full bank T-scores, between at least two groups for all characteristics, including orthosis level (F(2, 2) = [25.08], p < 0.001), paresis type (F(2, 2) = [27.59], p < 0.001), assistive device use (F(2, 2) = [286.45], p < 0.001), fewer falls in the prior 12 months (F(2, 2) = [15.02], p < 0.001) and number of comorbidities (F(2, 2) = [7.08], p < 0.001), as illustrated in Fig 3.

Strong, positive correlations were found between OPRO-M full item bank scores and those from OPUS-LEFS (r_s_ = 0.92, p < 0.001), LEFS (r_s_ = 0.87, p < 0.001), and PROMIS-PF (r_s_ = 0.87, p < 0.001). A moderate correlation was found between OPRO-M scores and PROMIS Ability to Participate in Social Roles and Activities scores (r_s_ = 0.57, p < 0.001) and weak correlations were found between OPRO-M scores and scores from other PROMIS instruments, including Fatigue (r_s_ = -0.36, p < 0.001), Pain Interference (r_s_ = -0.35, p < 0.001), Anxiety (r_s_ = -0.34, p < 0.001), Depression (r_s_ = -0.30, p < 0.001), and Sleep Disturbance (r_s_ = -0.18, p < 0.001).

## Discussion

Lower limb orthoses can compensate for the functional deficits associated with many neuromuscular and orthopedic health conditions, thereby improving a user’s overall mobility. Candidate items for the OPRO-M item bank were designed, through a rigorous qualitative process, to evaluate aspects of mobility that are most relevant to LLO use [19, 20]. In the current study, these candidate items were administered in a large national study and an IRT-based statistical analysis process [34] was employed to identify the items that are most effective for measuring orthotic mobility in clinical and research applications.

Results of the current study showed that 39 of the candidate items were unidimensional and locally independent, indicating that items measure a single primary trait (i.e., mobility) with minimal redundancy. OPRO-M items were also free of significant DIF, suggesting that the instrument can be used to measure mobility of LLO users without bias from variables that are generally unrelated to mobility. The 20- and 12-item short forms measured with high precision across a wide range of scores, and correlated highly with the full item bank, suggesting that the short forms can be used for most applications. Also, the mean reading level was less than 8^th^ grade level for the 20-item short form and less than 7^th^ grade level for the 12-item short form, indicating that OPRO-M short forms should be understandable to LLO users with 8^th^ grade or higher reading ability. There was no evidence of floor or ceiling effects, suggesting that few LLO users will receive scores at the lowest or highest extremes of the measurement scale.

The known-groups construct validity analysis indicated that OPRO-M scores can differentiate users by characteristics that are likely related to mobility, including number of comorbidities and assistive device use. The results of the convergent and divergent construct validity analyses provided evidence that OPRO-M correlates highly with PROMs that measure similar constructs (e.g., physical function), and does not correlate well with PROMs that measure dissimilar constructs (e.g., anxiety). While most of our hypotheses were confirmed, OPRO-M scores had weaker correlations with scores from PROMIS Fatigue and Pain Interference than anticipated. These findings suggest that LLO users’ mobility is quite distinct from their perceptions of fatigue or pain. Future research efforts could explore other methods of evaluating these constructs in order to provide a more wholistic view of LLO users’ functional abilities.

The effective range of measurement for the OPRO-M item bank is excellent, spanning nearly 2.75 SD above and below the mean. However, it did not reach our hypothesized range of 3 SD above and below the mean. Many of the candidate items that were removed through the analysis process were those that addressed low levels of mobility (e.g., Are you currently able to step sideways in both directions?) and high levels of mobility (e.g., Are you able to jump over low obstacles while running?). These items may have performed relatively poorly due to the small number of study participants in the present sample for whom these items would be applicable. Consequently, only one item in the OPRO-M 39-item bank addresses running. To broaden the effective range of measurement, future research efforts should include respondents at the extreme ends of the mobility continuum and expand OPRO-M by adding floor or ceiling items [55, 56]. Despite the limited number of items that address mobility at the extreme ends, the OPRO-M item bank, as well as the 20- and 12-item fixed length short forms measure with high reliability over a broad enough range that they are well-suited to evaluating mobility in clinical care and research. When measuring LLO users with very high or very low mobility, it may be preferable to create a custom short form that includes other items from the OPRO-M item bank that better target that range of mobility or use CAT administration.

OPRO-M also addresses the need for a PROM with items that are relevant to LLO users. OPRO-M items describe activities and movements that were identified through prior qualitative studies as being important to LLO users [13, 19]. Activities described in existing PROMs that were identified as less relevant to orthosis users, such as self-care tasks that involves upper limbs [56], bathing [12], and rolling over in bed [15], were not included in the OPRO-M item bank. Like other PROMs designed to measure aspects of physical functioning, most of the OPRO-M items describe walking and/or stepping. However, several of the contexts are unique from existing PROMs, including stepping over an extension cord, walking without catching one’s toes on the ground, and walking without looking down at the ground. Prior studies that examined LLO users’ perspectives concerning the importance of different types of activities support the emphasis of OPRO-M on ambulatory activities. Yang et al. [57] asked LLO users to rank a variety of activity types by importance to orthotic interventions and found that ambulatory activities were ranked highest. A focus group study conducted by van der Wilk et al. [18], similarly reported that walking was identified as being highly important and commonly performed while using an orthosis. We anticipate that the focus of OPRO-M on walking and stepping activities that are relevant to LLO users will resonate with patients responding to the items and clinicians providing their orthotic care.

The present study included a national sample with characteristics commonly observed in clinical rehabilitation settings. For example, the proportions of study participants who reported using different types of orthoses aligned closely with the types of devices orthotists reported providing to their patients in a national survey administered by the American Board for Certification in Orthotics, Prosthetics, and Pedorthics (ABC). The respective proportions reported in the current study and the ABC survey, respectively, were similar for AFOs (87.1%, 81.1%), KAFOs (9.6%, 14.5%), HKAFOs (0.7%, 0.3%), and FES devices (0.7%, 0.1%) [58]. The health conditions most frequently reported by participants in this study are also identified in the literature as being most relevant to LLO use, including stroke, spinal cord injury, orthopedic conditions, and Charcot-Marie-Tooth disease [59, 60]. These findings suggest that the reference sample for the OPRO-M item bank calibration is representative of the population of patients receiving orthotic care in clinical settings. The reference tables may therefore apply well to LLO users and can be used to interpret OPRO-M scores. Interpretation of OPRO-M scores is also made easier by a T-score that is centered on a sample of LLO users. When compared to existing item banks, like many of the PROMIS instruments that have scores centered on the U.S. general population [61], an OPRO-M T-score of 50 is the mean score of the current sample, which may be more understandable to patients and clinicians.

The IRT underpinnings of the OPRO-M item bank allow for flexibility and brevity in administration. The availability of multiple brief administration options may alleviate barriers that prevent clinicians from routinely using PROMs. A recent survey of prosthetists found that 36% would spend up to 5 minutes and 43% would spend up to 10 minutes for self-reported surveys during prosthetic appointments [62]. Although this study surveyed prosthetists about prosthetic care, many of these practitioners are dual certified as orthotists and may express similar feelings towards using PROMs during orthotic patient appointments. OPRO-M short forms could potentially fit into a 5-minute assessment window and minimize interruptions to patient care.

By addressing many of the barriers to use of PROMs (e.g., lack of perceived clinical value, challenges with interpretation, and excessive administrative burden), we expect that OPRO-M will be readily implemented into routine orthotic patient care. The Prosthetic Limb Users Survey of Mobility (PLUS-M) [63], a population-specific item bank that is similar to OPRO-M but developed for lower limb prosthesis users, is increasingly used in clinical practice to assess mobility outcomes in patients with lower limb amputation. Although PLUS-M is one of the more recent PROMs developed for prosthesis users, it has been translated into more than 20 languages [64], integrated into electronic health record software, and adopted as a primary clinical outcome measure by orthotic providers across the United States. As a result, researchers have been able to conduct retrospective studies of mobility-related outcomes in large, national samples of lower limb prostheses users [65–68]. PLUS-M has also increasingly been selected as a primary or secondary outcome in prosthetic clinical trials [69]. We anticipate that OPRO-M can be a similarly effective tool for orthotists and orthotic researchers interested in measuring mobility outcomes in clinical practice and research studies.

There were limitations to this study. First, participants included only those who used AFOs, KAFOs, HFAFOs, and FES devices. While many OPRO-M items may be applicable to individuals who use other types of LLOs (e.g., knee orthoses, foot orthoses), they were not designed for or tested with these LLO users. Further investigation would be required to assess OPRO-M’s suitability for these types of LLO users. Similarly, OPRO-M was designed explicitly for adult LLO users. As the use of a LLO may differ between adults and children, pediatric LLO users may benefit from the development of a PROM focused on activities that are more relevant to them (e.g., running with other children). Although we strived to identify a sample with characteristics that are representative of typical clinical populations, there may be health conditions (e.g., Parkinson’s disease) and personal attributes (e.g., non-white race) that were underrepresented. Future research could aim to collect more responses from diverse samples in order to update reference tables for score interpretation. Lastly, while initial evidence of validity was presented in the current study, additional evidence is needed for a comprehensive psychometric assessment. Future efforts should seek to establish additional evidence of construct validity by comparing OPRO-M scores to scores from mobility-related performance tests (e.g., Timed Up and Go Test, 10-meter Walk Test). Similarly, rigorous studies should be conducted to evaluate OPRO-M’s test-retest reliability by comparing OPRO-M scores from stable LLO users over consecutive administrations. Further, additional research will be required to examine whether OPRO-M is sensitive to the effects of orthotic interventions (i.e., different types of LLOs or levels of orthotic bracing).

## Conclusions

OPRO-M is a novel, IRT-based PROM for measuring mobility of lower limb orthosis users. It is calibrated to a national sample representative of a typical clinical population, includes activities and situations that are relevant to respondents, and can be administered quickly in clinical practice. The OPRO-M 12-item short form is recommended for most clinical and research applications. The 20-item short form measures with higher reliability across a broader range of mobility and is well-suited to evaluating populations with lower or higher levels of mobility. OPRO-M short forms and user guide are available at http://opro-m.org.

## Supporting information

S1 Dataset Dataset with participant characteristics and scores on patient-reported outcome measures that were used for construct validity analyses. OPRO-M: Orthotic Patient-Reported Outcomes–Mobility, LEFS: Lower Extremity Functional Scale, OPUS-LEFS: Orthotic and Prosthetic User’s Survey–Lower Extremity Functional Status, PROMIS: Patient-Reported Outcome Measure Information System, AFO: Ankle-foot orthosis(es), KAFO: Knee-ankle-foot orthosis(es).
(XLSX)
