# Correction: Development and initial validation of the Orthotic Patient-Reported Outcomes—Mobility (OPRO-M): An item bank for evaluating mobility of people who use lower-limb orthoses

**DOI:** 10.1371/journal.pone.0308468

**Published:** 2024-08-02

**Authors:** Geoffrey S. Balkman, Alyssa M. Bamer, Phillip M. Stevens, Eric L. Weber, Sara J. Morgan, Rana Salem, Dagmar Amtmann, Brian J. Hafne

In Table 1 the data in the columns n and % of rows "Age, Ethnicity, Race, and Orthosis type under Characteristic category are incorrect. Please see the correct Table 1 here.

**Table 1:** Sample characteristics.

*Characteristic*	*n*	*%*
*Gender*	* *	* *
Woman	520	50%
Man	514	50%
Other	2	<1%
*Age*		
18–39 years	117	11%
40–64 years	487	47%
65 or more years	432	42%
*Ethnicity*		
Hispanic or Latino	33	3%
Not Hispanic or Latino	948	92%
Unknown or prefer not to answer	55	5%
*Race* *		
American Indian or Alaskan Native	1	<1%
Asian	17	2%
Black or African-American	46	4%
Native Hawaiian or Pacific Islander	1	<1%
White	927	89%
Multiple races	18	2%
Other race	4	<1%
Unknown or prefer not to answer	22	2%
*Military status*		
Servicemember	2	<1%
Veteran	127	12%
Not Servicemember or Veteran	907	88%
*Geographical region*		
West	305	29%
Midwest	261	25%
Northeast	141	14%
South	329	32%
*Health condition* *		
Peripheral neuropathy	231	22%
Traumatic orthopedic LL injury	155	15%
Spinal cord injury	124	12%
Non-traumatic orthopedic LL condition	111	11%
Charcot-Marie-Tooth disease	97	9%
Stroke	90	9%
Multiple sclerosis	69	7%
Post-polio syndrome	65	6%
Muscular dystrophy	47	5%
Traumatic brain injury	43	4%
Non-traumatic spinal injury	39	4%
Other condition	177	17%
*Orthosis type*		* *
Unilateral AFO	649	63%
Bilateral AFO	283	27%
Unilateral KAFO	62	6%
Bilateral KAFO	14	1%
AFO and KAFO	12	1%
Unilateral HKAFO	4	<1%
Bilateral HKAFO	2	<1%
AFO and HKAFO	1	<1%
Unilateral FES	9	1%

*Respondents could select more than one option; LL: lower limb, AFO: ankle-foot orthosis, KAFO: knee-ankle-foot orthosis, HKAFO: hip-knee-ankle-foot orthosis, FES: functional electrical stimulation device.
